# Preliminary exploration of herbal tea products based on traditional knowledge and hypotheses concerning herbal tea selection: a case study in Southwest Guizhou, China

**DOI:** 10.1186/s13002-023-00645-w

**Published:** 2024-01-02

**Authors:** Xiaofeng Long, Sailesh Ranjitkar, Anna Waldstein, Huan Wu, Qingqing Li, Yanfei Geng

**Affiliations:** 1 College of Tea Science, Guizhou University, Guiyang, Guizhou China; 2 N.Gene Solution of Natural Innovation, Kathmandu, Nepal; 3 School of Developmental Studies and Applied Sciences, Lumbini Buddhist University, Lumbini, Nepal; 4 Resources Himalaya Foundation, Lalitpur, Nepal; 5 School of Anthropology and Conservation, University of Kent, Canterbury, UK

**Keywords:** Qianxinan, Herbal tea, Resource availability hypothesis, Versatility hypothesis, Diversification hypothesis, *Centella asiatica*

## Abstract

**Background:**

Herbal tea usually refers to “beverage plants that do not belong to the genus *Camellia*”, and it holds a significant historical legacy as a traditional beverage among specific regions and ethnic groups. In light of this, our research aims to investigate and analyze the traditional knowledge pertaining to herbal tea plants used by local people in the Qianxinan Buyi and Miao Autonomous Prefecture, Guizhou Province. We also initiated preliminary efforts to create tea products from herbal tea leaves using various processing techniques. Additionally, we attempted to test hypotheses to elucidate how local people select herbal tea plants.

**Methods:**

Data related to the use of herbal tea plants in this study were collected through semi-structured interviews and participatory observations in four villages in Qianxinan. Quantitative indicators, including the relative frequency of citation (RFC) and the relative importance (RI) value, were calculated, and the availability of plants was also evaluated. General linear model was performed to examine the relationship between the frequency of citation and resource availability, as well as the correlation between the relative frequency of citation and the relative importance, to test both the resource availability hypothesis and the versatility hypothesis. *Centella asiatica* tea was processed using techniques from green tea, black tea and white tea, with a preliminary sensory evaluation conducted.

**Results:**

A total of 114 plant species were documented as being used for herbal teas by local residents, representing 60 families and 104 genera. Of these, 61% of herbal tea plants were found growing in the wild, and 11 species were exotic plants. The family with the highest number of species was Asteraceae (20 species). The study identified 33 major medicinal functions of herbal tea, with clearing heat-toxin and diuresis being the most common functions. General linear model revealed a strong correlation (correlation coefficient of 0.72, *p* < 0.001) between the frequency of citation and plant availability, as well as a significant correlation (correlation coefficient of 0.63, *p* < 0.001) between RFC and RI. Under different processing conditions, the characteristics of *Centella asiatica* tea exhibited variations and were found to be suitable for consumption.

**Conclusion:**

The consumption of herbal tea serves as a preventive measure against common ailments for local residents. The resource availability hypothesis, diversification hypothesis and the versatility hypothesis were shown to provide some insight into “how and why local communities select plants for use.” Exotic herbal tea plants in the study area also possess valuable therapeutic properties. The processing and production of *Centella asiatica* herbal tea products hold promising prospects.

## Background

Chinese tea culture can be traced back to ancient times, boasting a remarkable antiquity. According to *The Classic of Tea* by Yu Lu in the Tang Dynasty (around year 780), tea has a history over 5000 years in China. The earliest tea drinking in Chinese history is associated with the emperor Shennong (茶之为饮, 发乎神农氏), who is credited with establishing Chinese agriculture and medicine. Shennong supposedly tested hundreds of wild herbs to see whether they had any therapeutic use. Multiple research reports show that flavonoids, alkaloids, phenols and other biologically active ingredients with immune-boosting characteristics are found in herbal teas [1, 2]. Herbal teas can even improve people’s mental health [3–6]. Additionally, herbal teas include polyphenols, saponins, oligosaccharides, selenium, zinc and other useful components and are an important source of antioxidants [7].

Typically, tea is regarded to be a beverage produced from the leaves and buds of the genus *Camellia* (sect. Thea, including *C. sinensis* var. *sinensis*, *C. sinensis* var. *assamica*, *C. sinensis* var. *dehungensis*, *C. taliensis*, *C. crassicolumna*). The term "herbal tea" is currently ambiguous but it generally refers to infusions made from the roots, stems, leaves, flowers, fruits and even whole plants of various plant species excluding those from the *Camellia* genus [8]. Herbal tea plants can be used alone or in conjunction with other herbs to make beverages. According to Traditional Chinese Medicine and other ethnomedical systems of China, internal heat can build up in the body, especially during the transitions between seasons [9]. People typically rely on herbal plants to prevent and treat ailments in their daily lives in places where medical clinics are underequipped [10, 11]. Preclinical research provides support for the use of herbal teas in the treatment of various illnesses [12]. For instance, the plant *Linum usitatissimum,* used to make traditional tea drinks “Emolientes and Emolienteros” from Peru, demonstrates the capacity to repair liver damage [13]. In Greece, chamomile tea (*Matricaria chamomilla*) may reduce the risk of thyroid cancer [14], while the legume tea plants *Aspalathus linearis* and *Cyclopia intermedia* from South Africa exhibit anti-mutagenic properties [15]. Similarly, the infusion of *Chrysanthemum morifolium*, a popular Asteraceae plant in Japan, has shown improvements in type II diabetes conditions [16]. Eagle tea, primarily produced in southwest China from the leaves of *Litsea coreana*, contains total flavonoid, which can lower serum triglycerides in rat blood and liver tissue as well as prevent lipid absorption [17]. Additionally, Dihydromyricetin found in vine tea (*Ampelopsis grossedentata*) has the ability to mitigate oxidative stress [18]. These findings underscore the potential of various herbal teas in the prevention and management of chronic metabolic disorders.

A wide variety of herbal tea plants can be found in township markets and are extensively consumed in 27 provinces of China [19]. Herbal tea beverages are highly popular in southern China due to their capacity to reduce internal heat and address various medical conditions. At present, there is not much research on herbal tea in Guizhou, except for eagle tea and Kuding tea, and research status regarding herbal tea uses is still not fully understood [20, 21]. Therefore, we performed the study in an effort to fill the gap left by the limited documentation of herbal tea in Guizhou.

In recent years, both in China and abroad, there has been a growing focus on researching herbal tea and developing new products using fresh plant leaves as the primary raw material [17, 22, 23]. Additionally, the traditional tea-making processes have seen enhancements. Steps such as fixation, withering, fermentation, rolling and drying are crucial in the tea processing industry [24]. These steps not only enhance the color, aroma and taste of tea but also significantly improve its flavor quality. Moreover, they increase the content of active ingredients in herbal tea. Currently, the processing of herbal tea primarily involves processes like evaporation, fermentation, rolling and drying [25–27], and it is in the early stages of being systematized. *Centella asiatica*, a plant belonging to the Apiaceae family, is widely used by locals in the study area for various purposes. Previous research has indicated that *Centella asiatica* is rich in a variety of phenolic compounds, such as rutin, quercetin and chlorogenic acid [26, 28], which are recognized for their antioxidant properties. Therefore, we aim to investigate the impact of different processing techniques on the quality of *Centella asiatica* herbal tea. This research will serve as a reference for the processing of other types of herbal teas.

In 2017, Gaoue et al. [29] synthesized existing research theories and pathways, reviewing and discussing 17 major ethnobotanical theories and hypotheses. More and more effort has been made to develop theories and hypotheses to promote ethnobotany as a hypothesis- and theory-driven discipline [30–32]. Hypotheses including resource availability hypothesis, diversification hypothesis and versatility hypothesis were put forward to address “how and why do local people select plants for use” [29]. The resource availability hypothesis states that a given plant is used because it has more accessibility or local abundance [33]. The versatility hypothesis predicts that people are more likely to retain knowledge, use and access to a plant that has a greater number of applications for humans [33, 34]. It is believed that plants that are used more tend to be plants with a large use category, which is measured by the relative importance (RI) of plants to reflect the utilization degree of a certain plant in the local community [34]. The diversification hypothesis is proposed to contribute to the understanding of the reasons why exotic species are incorporated into local medical systems which alien plants could help fill therapeutic gaps not filled by native plants [35]. Theoretical hypotheses could provide the basic structure of a discipline, help explain and predict phenomena, promote innovation and discovery and promote the further development of the discipline. Without a solid theoretical foundation, ethnobotanical research will lack direction and coherence, making it difficult to achieve lasting progress. Thus the present work also is intended to test these three hypotheses put forward by other ethnobotanists, as well as to discuss the possible selection strategies related to the use of herbal tea plants in China.

### Study area

**Fig. 1:**
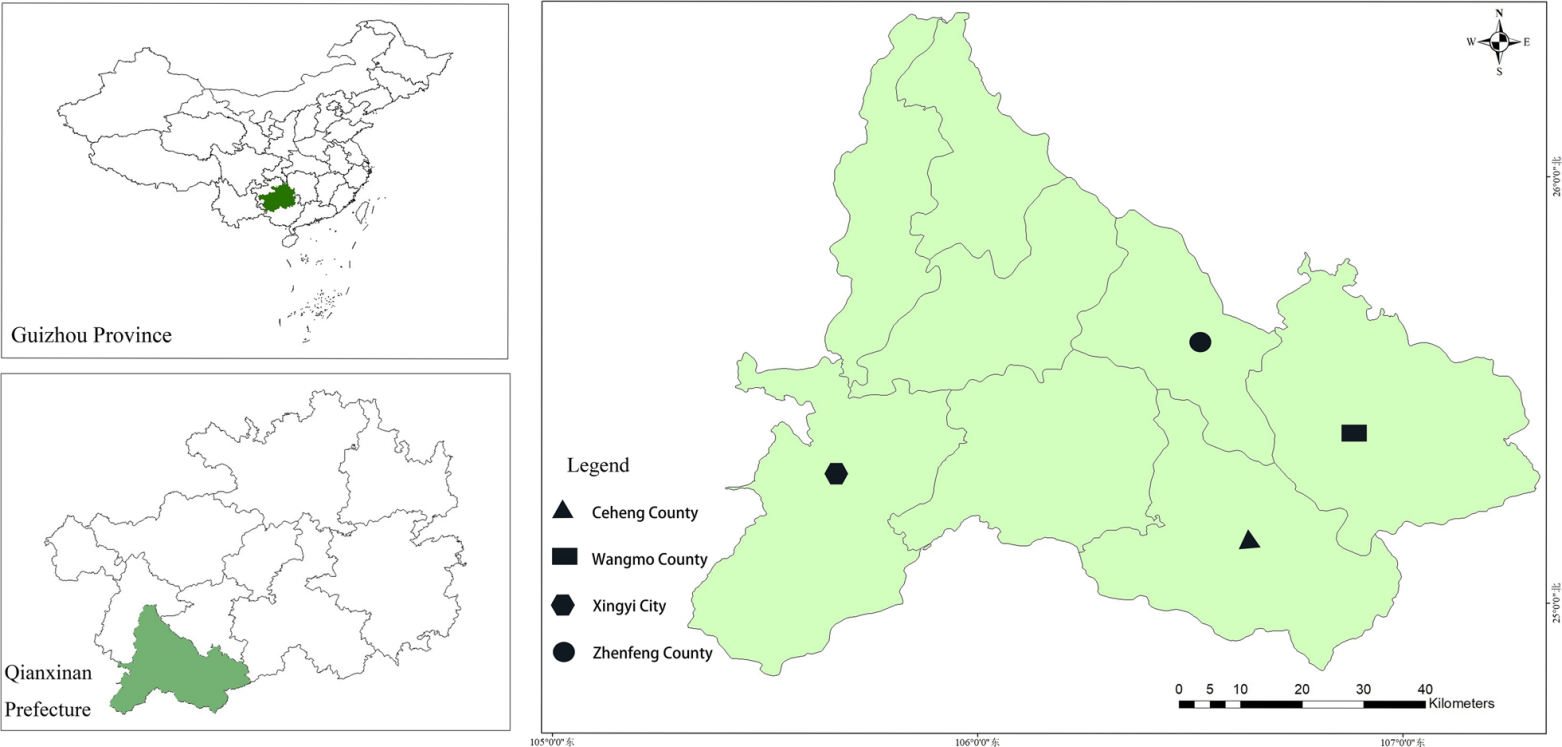
Study areas in Qianxinan Prefecture of Guizhou

Qianxinan Miao and Buyi prefecture (at 104° 35′–106° 32′ east longitude, 24° 38′–26° 11′ north latitude) is located in the southwest of Guizhou Province in Southern China. The total area of the prefecture is 16, 805 km^2^, accounting for 9.7% of the total area of the province. According to official statistics from 2019, the registered population of the prefecture reached more than 3,688,100. The prefecture has 35 ethnic minorities including Buyi, Miao, Hui and Han, among which Buyi has the largest population. The region has a humid subtropical monsoon climate, with an average annual temperature of 13.8–19.4 °C and an average annual rainfall of 1352.8 mm. The forest area of the prefecture is 1.010 million hm^2^, the forest area is 1,027,000 hm^2^, and the forest coverage rate reaches 61.17% [36]. Our research covered four counties/cities (Xingyi City, Zhenfeng County, Wangmo County and Ceheng County) with relatively concentrated ethnic minorities and rich traditional cultural diversity (Fig. 1).

## Methods

### Ethnobotanical survey

Ethnobotanical surveys on the utilization of herbal tea plants in Qianxinan Prefecture were carried out in the four villages in the studied counties from June 2020 to October 2021. After explaining the purpose of our study to the local community government and obtaining permission, the local community staff were invited to join us in the field survey as translators. Informants were selected by snowball sampling [37]. Altogether 110 informants (53 women and 57 men) aged between 14 and 86 were interviewed (Table 1), from four ethnic groups: Buyi, Miao, Yi and Mongolian. Semi-structured interview and participatory observation were used to collect information on herbal tea plants from selected informants [38]. Herbal tea plant specimens were collected in the field, and photographs of the plants were also taken while the informants were there.

**Table 1:** Statistics on the number of informants

Category	Number	%
*Gender*		
Male	57	51
Female	53	49
*Age group*		
≤ 30	7	6
31–50	27	25
51–70	62	56
≥ 71	14	13
*Ethnicity*		
Buyi	90	82
Miao	16	14
Yi	3	3
Mongolian	1	1

Using descriptions in the Flora of China (http://www.iplant.cn/) as a guide, herbal tea plant specimens were identified and cataloged to the herbal tea plants list. PuBiao network pharmacopoeia (http://www.zhongyoo.com/, https://db2.ouryao.com/) was used to confirm whether herbal tea plants were used for medicinal herbs. The exotic plant species were screened according to the “Alien Invasive Flora of China” (five volumes) [39–43], and their origin was determined by referring to the “Flora of China”.

### Data analysis

Relative frequency of citation (RFC) was used to show the local importance of each collected herbal tea plants [44]. The relative frequency of citation (RFC) formula is:

1 $${\text{RFC}}_{{\text{S}}} = \frac{{{\text{FC}}_{{\text{S}}} }}{N} = \frac{{\sum\limits_{{i = i_{1} }}^{iN} {{\text{UR}}_{i} } }}{N}$$

The frequency of citation (FC) refers to the number of informants mentioning the particular species, and *N* is the total number of informants, without considering the use categories of species [45]. Theoretically, the value range of this indicator is 0 < RFC < 1. The larger the index, the higher the importance of the species in the survey area.

Jaccard index (JI) is calculated to compare similarities in herbal tea uses between four counties (cities) [46]. The Jaccard Index formula is:

2 $${\text{JI}} = \frac{c}{{a + b + c}}$$

*a* is the number of plant species utilized only in region A; *b* is the number of plant species utilized only in region B; *c* is the number of plant species utilized in both regions.

The relative importance (RI) of the species was calculated according to the formula [47].

3 $${\text{RI}} = {\text{NUC}} + {\text{NT}}$$

The purpose of the RI is to investigate the types of use of a given species by respondents and the specific uses within each category, so that the “number of use categories” and “the number of types of uses” can be used to calculate the degree of use of the plant in a particular region or culture. NUC (number of use categories) is the quotient between the number of use types of a plant and the number of use types of the most used plant [46]; NT (number of types) is the quotient between the number of uses of the plant and the number of uses of the most important species.

Resource availability of herbal tea plants was assessed according to informants’ description and authors’ observation during fieldwork which was ranked by scoring one (most rare) to five (most common). General linear model was performed using SPSS (27.0.1) to analyze the correlation between the frequency of citation and resource availability, as well as the correlation between relative frequency of citation and the relative importance, to test both the resource availability hypothesis and the versatility hypothesis [33, 34, 48]. The correlationship image was created using the Origin Por 2022 software (Version 9.9) [49].

## Preliminary product research

### Materials

Fresh leaves of *Centella asiatica* were collected from *Centella asiatica* experimental site of West Campus of Guizhou University in April 2023. Fresh samples were processed into green tea, black tea and white tea, respectively.

### Processing

The fresh leaves from the same batch were combined and then divided into three equal samples, each weighing one kilogram. Then each sample was processed into tea samples of *Centella asiatica* green tea, *Centella asiatica* black tea and *Centella asiatica* white tea according to green tea, black tea and white tea processing standard [50]. The processing of *Centella asiatica* green tea primarily involves evaporating, rolling and drying, the processing of *Centella asiatica* black tea follows the steps of withering, rolling, fermenting and drying, while the processing of *Centella asiatica* white tea consists of withering and drying.

### Sensory evaluation

The appearance of tea product, appearance of tea liquid, aroma, taste and tea leaves quality characteristics of *Centella asiatica* tea were evaluated according to the national standard (GB/T 23776-2018 [51], GB/T 14487-2017 [52]) and “Tea sensory evaluation Terms” (T/CTSS 58-2022) [52, 53].

## Results

### Diversity

In total, 114 species of herbal tea plants representing 60 families and 104 genera were recorded in Qianxinan (Table 2), and they were widely distributed in various families. The majority of herbal tea plant species in the survey area were found along roadsides (48 species) and in fields (29 species). They were also present under the forests, near houses and in grasslands. The Asteraceae family had the highest number of plant species (20 species), followed by the Lamiaceae family, which included seven herbal tea plant species. The Rosaceae, Apiaceae and Fabaceae families each had five species of herbal tea plants, while other families had fewer species.

**Table 2:** The inventory of herbal tea plants used by local people in Southwest Guizhou, China

Family	Scientific name	Part of plant used	Life form	Collecting locations	Collecting time	Processing methods	Therapeutic use	Taboo	Voucher number	Habitat	RFC	RI
Acanthaceae	*Dicliptera chinensis* (L.) Juss	Whole plant	Herb	Streamside, Roadside	All year round	Decoction	Clear heat-toxin, diuresis		–*	W	0.009	0.83
Altingiaceae	*Liquidambar formosana* Hance	Leaf, Root, Fruit	Tree	Village, Understory	Spring, Autumn	Decoction	Eliminate wind and resolve dampness , circulate blood and relax tendons, circulate blood and resolve stasis		QXN083*	W	0.009	1.31
Amaranthaceae	*Amaranthus tricolor* L	Root, Fruit, Whole plant	Herb	Field	Summer, Autumn	Decoction	Brighten the eyes, diuresis, promote bowel movements		QXN050	C/W(E)	0.026	0.97
	*Chenopodium album* L	Whole plant	Herb	Roadside, Wilderness, Field	All year round	Sun drying, Decoction	Stop abdominal pain, invigorate spleen to promote digestion		QXN057	W	0.009	0.83
Apiaceae	*Centella asiatica* (L.) Urban	Whole plant	Herb	Mountains and plains, Understory	Summer	Soak in hot water, Blanching, Decoction	Moisten the lung and relieve cough, circulate blood and resolve stasis , diminish inflammation		QXN065*	W	0.388	2.00
	*Cryptotaenia japonica* Hassk	Whole plant	Herb	Grass, Brookside, Understory	All year round	Decoction	Clear heat and eliminate dampness, moisten the lung and relieve cough, remove toxins and circulate blood, hemostatis		QXN089	W	0.078	1.31
	*Hydrocotyle sibthorpioides* Lam	Whole plant	Herb	Grass, Ditchside	Spring, Summer	Soak in hot water	Clear heat and eliminate dampness, soften hardness and dissipate masses, circulate blood and resolve stasis		QXN082*	W	0.026	1.66
	*Oenanthe javanica* (Bl.) DC	Whole plant	Herb	Marsh, Ditchside, Behind the house	All year round	Soak in hot water, Decoction	Clear heat and eliminate dampness, hemostatis, lower blood pressure		QXN001	C/W	0.078	1.51
	*Peucedanum medicum* Dunn	Root	Herb	Field, Mountains and plains	Spring	Decoction	Clear heat-toxin, circulate blood and resolve stasis		QXN014	C	0.112	0.83
Araceae	*Sauromatum horsfieldii* Miquel	Stem	Herb	Understory, Behind the house	All year round	Using fresh herb, Sun drying, Decoction	Soften hardness and dissipate masses, circulate blood and resolve stasis		QXN072	C	0.009	0.49
Araliaceae	*Eleutherococcus trifoliatus* (Linnaeus) S. Y. Hu	Root, Leaf	Shrub	Village, Roadside, Understory	All year round	Decoction	Eliminate wind and resolve dampness, resolve swelling, alleviate pain	Cold	QXN076	C	0.069	1.17
	*Eleutherococcus nodiflorus* (Dunn) S. Y. Hu	velamen	Shrub	Understory, Roadside	Spring, Autumn	Decoction, Soak medicated wine	Eliminate wind and resolve dampness , strengthen the sinews and bones, soften hardness and dissipate masses		QXN013*	C	0.026	0.63
	*Eleutherococcus senticosus* (Ruprecht & Maximowicz) Maximowicz	velamen	Shrub	Village, Behind the house	Summer, Autumn	Decoction, Soak medicated wine	Eliminate wind and resolve dampness , nourish liver, strengthen muscles and bones		–*	W	0.017	1.17
Asphodelaceae	*Hemerocallis citrina* Baroni	Fruit, Flower	Herb	Mountains and plains, Wilderness, Behind the house	Spring, Summer	Sun drying, Soak in hot water	Diuresis, hemostatis		–*	C	0.026	1.17
Asteraceae	*Acmella paniculata* (Wallich ex Candolle) R. K. Jansen	Whole plant	Herb	Field, Brookside	Spring, Summer	Sun drying, Soak in hot water	Clear heat-toxin, brighten the eyes	Cold, slightly toxic	QXN091*	W(E)	0.009	0.63
	*Adenostemma lavenia* (L.) O. Kuntze	Whole plant	Herb	Understory, Grass, Brookside, Field, Wilderness	Spring	Sun drying, Decoction	Clear heat-toxin, diuresis	Cold	QXN075	W	0.026	0.49
	*Artemisia argyi* Lévl. et Van	Leaf	Herb	Grass, Brookside, Fieldborders, Roadside	Autumn, Winter	Sun drying, Decoction	Clear heat-toxin, nourish liver		QXN049*	W	0.009	1.66
	*Aster indicus* L	Aboveground part	Herb	Fieldborders, Ditchside, Streamside, Wilderness, Roadside	Spring, Summer, Autumn	Decoction	Moisten the lung and relieve cough, clear heat and eliminate dampness, alleviate pain		QXN009	W	0.086	0.97
	*Bidens pilosa* L	Whole plant	Herb/Shrub	Understory, Brookside, Grass	Autumn	Decoction, Soak in hot water	Refresh oneself, alleviate pain		QXN028*	W	0.034	1.11
	*Bidens bipinnata* L	Whole plant	Herb	Mountains and plains, Grass, Understory	Summer, Autumn	Sun drying, Decoction	Alleviate pain, dissipate cold and eliminate dampness		QXN017*	W	0.009	0.63
	*Blumea balsamifera* (L.) DC	Whole plant	Herb	Roadside, Ditchside	All year round	Decoction	Treat dysentery, invigorate spleen to promote digestion, clear heat-toxin		QXN039*	W(E)	0.009	0.83
	*Chrysanthemum indicum* Linnaeus	Leaf, Flower, Whole plant	Herb	Village, Roadside, Wilderness	All year round	Sun drying, Brewing, Soak in hot water	Stop malaria, clear heat-toxin, circulate blood, hemostatis		QXN002*	W(E)	0.052	1.17
	*Crassocephalum crepidioides* (Benth.) S. Moore	Whole plant	Herb	Understory, Grass, Field, Roadside	All year round	Soak in hot water	Clear heat-toxin, treat sores		QXN054	W	0.069	0.97
	*Eclipta prostrata* (L.) L	Whole plant	Herb	Understory, Streamside, Roadside	Summer, Autumn	Sun drying, Decoction	Clear heat-toxin, invigorate spleen to promote digestion, diuresis		QXN010*	W	0.017	1.31
Asteraceae	*Emilia prenanthoidea* DC	Whole plant	Herb	Grass, Understory, Ditchside, Village, Brookside	Spring	Sun drying, Decoction	Clear heat-toxin, circulate blood and resolve stasis		QXN022*	W	0.009	0.49
	*Erigeron annuus* (L.) Pers	Whole plant	Herb	Ditchside, Roadside, Understory, Mountains and plains	Summer, Autumn	Using fresh herb, Sun drying, Decoction	Clear heat and eliminate dampness, soften hardness and dissipate masses	Cold	QXN077	W	0.009	0.97
	*Erigeron breviscapus* (Vant.) Hand. -Mazz	Whole plant	Herb	Grass, Roadside, Field, Brookside	Spring, Summer, Autumn	Decoction	Clear heat-toxin, soften hardness and dissipate masses, lower blood pressure		QXN086*	W	0.026	0.49
	*Ixeris polycephala* Cass	Whole plant	Herb	Understory, Streamside	All year round	Decoction	Clear heat-toxin, soften hardness and dissipate masses, nourish liver, brighten the eyes		QXN021	W	0.069	1.17
	*Pseudognaphalium affine* (D. Don) Anderberg	Stem, Leaf	Herb	Grass, Rcefield	Spring	Soak in hot water	Clear heat-toxin, eliminate wind and resolve dampness, lower blood pressure		QXN064	W	0.009	1.11
	*Senecio scandens* Buch.-Ham. ex D. Don	Whole plant	Herb	Roadside, Wilderness	All year round	Decoction	Clear heat-toxin, stop malaria, diminish inflammation and alleviate pain		QXN030*	W(E)	0.017	0.77
	*Sonchus oleraceus* L	Leaf	Herb/Shrub	Wilderness, Roadside, Brookside, Mountains and plains	Summer	Decoction, Blanching, Soak in hot water	Dissipate cold and eliminate dampness, alleviate pain, refresh oneself		QXN019	W	0.043	1.17
	*Sonchus wightianus* DC	Whole plant	Herb	Brookside, Fieldborders, Roadside	Summer	Decoction, Blanching, Soak	Circulate blood and resolve stasis, hemostatis, tonify		QXN040	W(E)	0.129	0.83
	*Taraxacum mongolicum* Hand.-Mazz	Whole plant	Herb	Mountains and plains, Understory	All year round	Using fresh herb, Sun drying, Decoction	Clear heat-toxin, diminish inflammation		QXN016*	W	0.172	1.66
	*Youngia japonica* (L.) DC	Whole plant	Herb	Roadside, Field	Spring	Stir frying, Decoction	Stop diarrhea, stop malaria, clear heat-toxin		QXN059	W(E)	0.19	0.83
Berberidaceae	*Berberis sargentiana* Schneid	Root	Shrub	Mountains and plains, Roadside, Understory	All year round	Decoction	Clear heat-toxin, diminish inflammation and antisepsis		–*	W	0.026	0.49
	*Mahonia fortunei* (Lindl.) Fedde	Whole plant	Shrub	Rinchu, Roadside, Brookside	All year round	Decoction	Clear heat-toxin, tonify	People with weak and cold physique should not be use.	QXN042*	C/W	0.017	0.83
Cabombaceae	*Brasenia schreberi* J. F. Gmel	Whole plant	Herb	Pond, Brookside, Marsh	Summer, Autumn	Using fresh herb, Decoction	Clear heat-toxin, soften hardness and dissipate masses	Cold	–	W	0.052	0.83
Campanulaceae	*Lobelia nummularia* Lam	Whole plant	Herb	Rcefield	All year round	Using fresh herb, Sun drying, Decoction	Eliminate wind and resolve dampness, traumatic injury		QXN046	W	0.043	0.49
Caprifoliaceae	*Lonicera similis* Hemsl	Flower	Vine	Mountains and plains, Streamside, Rinchu	Spring	Stewing, Sun drying, Brewing	Clear heat-toxin, diminish inflammation, resolve swelling		QXN007*	W	0.371	1.80
	*Platycodon grandiflorus* (Jacq.) A. DC	Root	Herb	Brush, Behind the house	Spring, Autumn	Decoction	Moisten the lung and relieve cough, diminish inflammation		QXN008*	C/W	0.009	1.17
	*Viburnum foetidum* Wall	Fruit	Shrub	Mountains and plains, Brush	Autumn	Using fresh herb, Soak in hot water	Clear heat-toxin, moisten the lung and relieve cough, diminish inflammation, repair broken sinews and bones, stop diarrhea		QXN047	W	0.026	1.31
Caryophyllaceae	*Stellaria aquatica* (L.) Scop	Whole plant	Herb	Ditchside, Field	Spring	Soak in hot water	Clear heat and relieve strangury, soften hardness and dissipate masses, unblock meridians and increase lactation		QXN085	C/W(E)	0.052	1.11
Celastraceae	*Gymnotheca chinensis* Decne	Whole plant	Herb	Ditchside, Rinchu	Autumn, Winter	Soak in hot water, Decoction	Invigorate spleen to promote digestion, remove toxins		QXN015	W	0.034	0.83
Clusiaceae	*Hypericum beanii* N. Robson	Root, Leaf, Bud	Shrub	Understory, Streamside, Mountains and plains	Summer	Using fresh herb, Sun drying, Decoction	Nourish liver, circulate blood hemostatis	Cold	QXN078	W	0.009	0.49
Commelinaceae	*Commelina communis* L	Whole plant	Herb	Brush, Roadside	Summer, Autumn	Decoction	Resolve swelling, clear heat-toxin		QXN012*	C/W	0.009	0.49
Convolvulaceae	*Argyreia pierreana* Bois	Stem, Leaf	Vine	Roadside, Brush	Autumn	Decoction	Traumatic injury, eliminate wind and resolve dampness, alleviate pain		–*	W	0.026	1.66
Crassulaceae	*Phedimus aizoon* (Linnaeus) 't Hart	Whole plant	Herb	Field, Wilderness	All year round	Decoction	Circulate blood and resolve stasis, alleviate pain, lower blood pressure		QXN025*	C/W	0.009	0.63
Cucurbitaceae	*Gynostemma pentaphyllum* (Thunb.) Makino	Whole plant	Herb	Roadside, Rinchu	Autumn, Winter	Decoction, Brewing	Clear heat-toxin, soften hardness and dissipate masses	Cold	QXN035*	W	0.017	0.97
	*Solena heterophylla* Lour	Root	Vine	Rinchu, Mountains and plains, Roadside	Autumn	Using fresh herb, Sun drying, Decoction	Diminish inflammation, moisten the lung and relieve cough, lower blood pressure	Patients with asthenia-cold disease and pregnant women should take it with caution.	QXN087	W	0.009	0.49
Cyperaceae	*Cyperus rotundus* L	Stem	Herb	Wilderness, Ditchside, Roadside	Spring, Summer, Autumn	Soak in hot water	Qi regulate, alleviate pain and regulate menstruation		–*	W(E)	0.009	0.83
Equisetaceae	*Equisetum ramosissimum* Desf	Aboveground part	Herb	Village, Roadside	All year round	Decoction	Reduce blood lipid, diuresis, lower blood pressure	Whole plant is toxic.	QXN034*	W	0.009	0.91
Fabaceae	*Crotalaria ferruginea* Grah. ex Benth	Whole plant	Vine	Mountains and plains, Understory	Autumn, Winter	Decoction	Circulate blood and relax tendons, remove alcoholic toxins, stop diarrhea		–	W	0.034	0.63
	*Glycyrrhiza uralensis* Fisch	Stem, Root	Herb	Brookside, Mountains and plains, Grass	Spring, Autumn	Decoction	Tonify, clear heat-toxin, moisten the lung and relieve cough, alleviate pain	Patients with high blood pressure and swelling should not use it.	–*	C	0.009	1.11
	*Pueraria montana* (Loureiro) Merrill	Root	Shrub	Brookside, Pond	Summer, Autumn	Sun drying, Soak in hot water	Nourish liver, diuresis, stop malaria		QXN036*	C/W(E)	0.009	1.11
	*Senna occidentalis* (Linnaeus) Link	Seed, Root, Leaf	Shrub/Tree	Field, Mountains and plains, Roadside	Summer, Autumn	Stir frying, Decoction	Clear heat-toxin, diuresis, soften hardness and dissipate masses	The whole plant is toxic, and users should take reasonable dose.	QXN031	W	0.009	1.46
	*Sophora davidii* (Franch.) Skeels	Root, Leaf, Flower, Seed	Herb	Mountains and plains, Rinchu, Wilderness	All year round	Stir frying, Decoction	Warm and tonify kidney Yang, diminish inflammation, diuresis		QXN066	W	0.026	0.63
Fagaceae	*Lithocarpus polystachyus* Rehder	Leaf	Tree	Village, Rinchu	All year round	Mash, Filter, Decoct, Sun drying, Brewing	Clear heat-toxin, lower blood pressure, reduce blood lipid, regulate blood sugar level, warm and tonify kidney Yang		QXN053	W	0.086	1.46
Hypericaceae	*Hypericum japonicum* Thunb.ex Murray	Whole plant	Herb	Understory, Mountains and plains	All year round	Sun drying, Soak in hot water	Clear heat-toxin, hemostatis, soften hardness and dissipate masses		–*	W	0.009	0.49
Lamiaceae	*Ajuga decumbens* Thunb	Whole plant	Herb	Field, Grasscluster, Roadside	Spring	Soak in hot water	Antisepsis and remove toxins, treat epidemic toxin dysentery	Cold	–	W	0.009	0.49
	*Callicarpa macrophylla* Vahl	Leaf, Root	Herb	Streamside, Roadside	Summer	Sun drying, Decoction	Clear heat-toxin, nourish liver		QXN045*	C/W	0.009	0.49
	*Leonurus japonicus* Houttuyn	Whole plant	Herb	Grass, Streamside, Roadside	Summer	Using fresh herb, Sun drying, Soak in hot water	Nourish liver, brighten the eyes	Pregnant women are prohibited from use.	QXN071*	W	0.009	0.97
	*Mentha canadensis* Linnaeus	Aboveground part	Herb	Mountains and plains, Roadside, Village, Wilderness, Behind the house	Summer	Air drying	Dissipate cold and eliminate dampness, tonify the stomach	P regnant women are prohibited from use.	QXN051*	C/W	0.026	0.97
	*Perilla frutescens* (L.) Britt	Stem, Leaf, Fruit	Herb	Ditchside, Field	Summer, Autumn	Using fresh herb, Decoction	Clear heat-toxin, clear the throat, nourish liver		QXN027*	C	0.009	0.83
	*Prunella vulgaris* L	Whole plant	Shrub/Tree	Understory, Brush, Roadside	Summer, Autumn	Using fresh herb, Sun drying, Decoction	Circulate blood and resolve stasis, soften hardness and dissipate masses		QXN005*	C	0.026	0.49
	*Salvia prionitis* Hance	Whole plant	Herb	Behind the house, Mountains and plains	Spring, Summer	Sun drying, Decoction	Regulate menstruation, diuresis, clear heat-toxin		–	C	0.009	0.49
Lardizabalaceae	*Akebia trifoliata* (Thunb.) Koidz	Root, Stem, Fruit	Vine	Mountains and plains	All year round	Decoction	Clear heat and eliminate dampness, circulate blood and relax tendons, soften hardness and dissipate masses		QXN090*	W	0.009	0.97
	*Holboellia latifolia* Wall	Root, Seedcase	Vine	Mountains and plains, Rinchu, Brush	Summer, Autumn	Decoction	Diuresis, circulate blood and relax tendons, eliminate wind and resolve dampness		QXN029*	W	0.017	0.97
	*Sargentodoxa cuneata* (Oliv.) Rehd. et Wils	Root, Stem	Vine	Brush, Rinchu	Autumn, Winter	Sun drying, Decoction	Clear heat-toxin, eliminate wind and resolve dampness, alleviate pain		QXN063*	W	0.017	0.63
Lauraceae	*Litsea lancilimba* Merr	Fruit	Tree	Rinchu	Autumn	Sun drying, Soak in hot water	Dissipate cold and eliminate dampness, alleviate pain		–	W	0.026	0.83
Liliaceae	*Lilium brownii* F. E. Brown ex Miellez	Stem	Herb	Understory, Mountains and plains, Village, Behind the house	Autumn	Using fresh herb, Decoction	Moisten the lung and relieve cough, diuresis		QXN088*	C/W	0.009	0.83
Lythraceae	*Punica granatum* L	Leaf	Shrub/Tree	Behind the house	Autumn	Soak in hot water	Stop diarrhea, invigorate spleen to promote digestion		QXN070*	C	0.026	0.97
Malvaceae	*Firmiana simplex* (L.) W. Wight	Seed, Stem, Flower, Leaf, Fruit	Tree	Behind the house	Summer, Autumn	Drying, Decoction	Clear heat-toxin, lower blood pressure, hemostatis, reduce blood lipid		QXN079*	C	0.009	1.11
Melanthiaceae	*Paris polyphylla* Smith	Flower	Herb	Ditchside, Rinchu, Brush, Roadside	Autumn	Using fresh herb, Sun drying, Decoction	Clear heat-toxin, soften hardness and dissipate masses, nourish liver	It is forbidden to take the medicine if the body is weak or no heat-toxin; pregnant women are not allowed to take it.	–*	C	0.009	0.63
Melastomataceae	*Melastoma malabathricum* Linnaeus	Whole plant, Root	Shrub	Mountains and plains, Understory, Ditchside	Summer	Using fresh herb, Soak in hot water	Clear heat and eliminate dampness, soften hardness and dissipate masses, circulate blood, hemostatis		–*	W	0.017	0.97
Moraceae	*Morus alba* L	velamen, Fruit	Shrub/Tree	Behind the house, Field	Spring, Summer	Soak in hot water	Clear heat-toxin, clear lung heat and moisten dryness, nourish liver, brighten the eyes		QXN069*	C	0.017	0.97
Musaceae	*Musa basjoo* Sieb. et Zucc	Root, Flower, Root	Herb	Behind the house, Field	Summer, Autumn	Decoction, Soak in hot water	Clear heat-toxin, moisten dryness and alleviate thirst, diuresis		QXN052	C	0.017	1.11
Myrtaceae	*Psidium guajava* L	Leaf	Tree/Shrub	Wilderness, Field	Summer	Sun drying, Decoction	Stop abdominal pain, stop diarrhea, hemostatis, tonify the stomach		QXN018*	C	0.026	0.97
Oleaceae	*Ligustrum expansum* Rehder	Leaf	Shrub	Streamside, Roadside	Spring, Summer	Soak in hot water	Clear heat-toxin , tonify, nourish liver, brighten the eyes, lower blood pressure		QXN056*	C/W	0.086	1.11
Onagraceae	*Oenothera rosea* L'Her. ex Ait	Root	Herb	Roadside, Brookside, Behind the house, Wilderness, Grass, Ditchside	Summer	Decoction	Diminish inflammation, lower blood pressure		QXN026	W(E)	0.009	0.97
Orchidaceae	*Cymbidium aloifolium* (L.) Sw	Whole plant	Epiphyte	Rinchu, Brush, Streamside	Summer	Decoction	Clear lung heat and moisten dryness		–	C	0.009	0.34
Oxalidaceae	*Oxalis corniculata* L	Whole plant	Herb	Brookside, Roadside, Fieldborders, Wilderness, Understory	All year round	Decoction	Clear heat and eliminate dampness, circulate blood, hemostatis, soften hardness and dissipate masses		QXN020*	W	0.026	0.63
Piperaceae	*Peperomia pellucida* (L.) Kunth	Whole plant	Herb	Understory, Wetland, Behind the house	Summer, Autumn	Soak in hot water	Circulate blood hemostatis, clear heat-toxin		–	W	0.009	0.49
Plantaginaceae	*Plantago asiatica* L	Whole plant	Herb	Grass, Ditchside, Wetland, Fieldborders, Roadside, Village	Summer	Decoction	Clear heat-toxin , diuresis		QXN023*	W	0.216	1.26
Poaceae	*Imperata cylindrica* (L.) Beauv	Stem	Herb	Mountains and plains, Roadside	Summer	Sun drying, Soak in hot water	Clear heat and reduce internal heat , moisten dryness and alleviate thirst, diuresis		QXN073*	C/W	0.026	0.83
	*Lophatherum gracile* Brongn	Root, Leaf	Herb	Brookside, Grass	Spring, Autumn	Soak in hot water	Hemostatis, diuresis	Those with physical weakness and pregnant women are forbidden to take it.	QXN080*	C	0.009	0.63
Polygonaceae	*Pleuropterus multiflorus* (Thunb.) Nakai	Root	Herb	Understory	Summer	Using fresh herb, Sun drying, Decoction	Clear heat and eliminate dampness, nourish liver, brighten the eyes, circulate blood and relax tendons		QXN024*	W	0.052	0.97
	*Persicaria capitata* (Buch. -Ham. ex D. Don) H. Gross	Whole plant	Herb	Brush, Mountains and plains, Understory, Behind the house	Autumn, Winter	Sun drying, Soak in hot water	Stop malaria, promote bowel movements		QXN074	C/W	0.017	0.49
	*Persicaria chinensis* (L.) H. Gross	Aboveground part	Herb	Mountains and plains, Wetland	Summer	Sun drying, Soak in hot water	Clear heat and eliminate dampness, circulate blood and resolve stasis, alleviate pain		QXN061*	W	0.026	0.63
Polypodiaceae	*Lepisorus carnosus* (Wall. ex J. Sm.) C. F. Zhao, R. Wei & X. C. Zhang	Whole plant	Fern	Understory	Autumn, Winter	Decoction, Soak in hot water	Moisten the lung and relieve cough, circulate blood and resolve stasis, clear heat-toxin		–	W	0.009	0.63
Portulacaceae	*Portulaca oleracea* L	Aboveground part	Herb	Garden, Farmland, Roadside	Spring, Summer	Blanching, Sun drying, Decoction	Soften hardness and dissipate masses, diminish inflammation, moisten dryness and alleviate thirst, diuresis		QXN043*	W	0.009	0.77
Primulaceae	*Ardisia crispa* (Thunb.) A. DC	Root, Leaf	Shrub	Valley, Understory	All year round	Sun drying, Decoction	Circulate blood and resolve stasis, hemostatis		QXN032*	C	0.026	0.49
	*Ardisia mamillata* Hance	Whole plant	Shrub	Fieldborders	Summer, Autumn	Airing, Soak in hot water medicated wine	Clear heat-toxin , alleviate pain, diminish inflammation		–	C/W	0.017	0.49
	*Myrsine africana* L	Stem, Leaf	Shrub	Mountains and plains, Understory	Summer	Decoction	Clear heat and eliminate dampness, circulate blood and relax tendons	The fruit is poisonous to animals.	QXN062	C/W	0.009	0.63
Ranunculaceae	*Clematis florida* Thunb	Root, Whole plant	Vine	Field, Roadside, Streamside	All year round	Decoction	Diuresis, promote bowel movements, alleviate pain		QXN037*	W	0.009	0.63
Rosaceae	*Agrimonia pilosa* Ldb	Whole plant	Shrub	Mountains and plains, Roadside	Autumn	Soak in hot water	Invigorate spleen to promote digestion, stop diarrhea		QXN055*	W	0.026	0.97
	*Eriobotrya japonica* (Thunb.) Lindl	Leaf, Flower	Shrub	Grass, Brookside, Roadside	Summer	Sun drying, Decoction	Clear heat-toxin, invigorate spleen to promote digestion, circulate blood and resolve stasis, hemostatis		QXN068*	W	0.034	0.83
	*Potentilla griffithii* Hook. f	Root	Herb	Grass, Field	Summer, Autumn	Soak in hot water	Invigorate spleen to promote digestion, alleviate pain		–	C/W	0.017	0.49
	*Pyracantha fortuneana* (Maxim.) Li	Fruit, Root, Leaf	Herb	Streamside, Roadside, Grass, Brush	Summer, Autumn	Using fresh herb, Soak in hot water	Hemostatis, stop malaria, stop abdominal pain, tonify		QXN011	W	0.069	1.11
	*Rosa roxburghii* Tratt	Fruit, Leaf	Tree	Roadside, Behind the house	All year round	Using fresh herb, Soak in hot water	Moisten the lung and relieve cough		QXN081	C	0.052	0.97
Sabiaceae	*Sabia parviflora* Wall. ex Roxb	Stem, Leaf	Vine	Field, Streamside, Rinchu, Brush	Summer, Autumn	Using fresh herb, Sun drying, Decoction, Brewing	Clear heat and eliminate dampness, nourish liver		–	W	0.026	0.97
Sapindaceae	*Cardiospermum halicacabum* L	Whole plant	Vine	Field, Brush, Roadside	Summer, Autumn	Soak in hot water	Clear heat and reduce internal heat, remove toxins, soften hardness and dissipate masses	Cold	–	C/W	0.009	0.97
Saururaceae	*Houttuynia cordata* Thunb	Whole plant	Herb	Ditchside, Streamside, Behind the house	Summer	Using fresh herb, Soak in hot water	Clear heat-toxin, resolve abscesses and dissipate boils, diuresis		QXN004*	C/W	0.483	1.60
Scrophulariaceae	*Buddleja officinalis* Maxim	Flower	Shrub	Field, Brookside, Village, Brush	Spring	Sun drying, Decoction	Clear heat-toxin, eliminate dampness, brighten the eyes	Caution should be taken if the eye disease is Yang deficiency and internal cold.	QXN058*	W	0.017	1.11
Selaginellaceae	*Selaginella tamariscina* (P. Beauv.) Spring	Whole plant	Fern	Mountains and plains	All year round	Decoction	Regulate menstruation, circulate blood and transform stasis		QXN033	W	0.009	0.49
Solanaceae	*Lycium chinense* Miller	Fruit	Shrub	Field, Wilderness, Roadside, Village, Behind the house	Autumn	Using fresh herb, Decoction	Tonify, nourish liver, brighten the eyes	Drink moderately.	QXN084*	C/W	0.009	0.83
	*Solanum nigrum* L	Aboveground part	Herb	Fieldborders, Wilderness, Village	Spring, Summer	Soak in hot water, Decoction	Circulate blood, hemostatis, clear heat-toxin	The leaves contain a lot of alkaloids, which must be fully cooked.	QXN003*	W	0.31	1.31
Solanaceae	*Solanum pseudocapsicum* L	Root	Shrub	Understory, Roadside, Wilderness	All year round	Sun drying, Decoction	Diminish inflammation and remove toxins, alleviate pain	Whole plant is toxic.	QXN044	W	0.009	1.11
	*Solanum violaceum* Ortega	Fruit, Root	Shrub	Fieldborders, Roadside	All year round	Using fresh herb, Sun drying, Decoction	Circulate blood and resolve stasis, hemostatis, soften hardness and dissipate masses	Toxic, should not overdose	QXN038	C/W(E)	0.009	0.49
Thelypteridaceae	*Cyclosorus acuminatus* (Houtt.) Nakai	Stem, Whole plant	Fern	Streamside, Rinchu	Summer, Autumn	Sun drying, Decoction	Clear heat-toxin, eliminate wind and resolve dampness, invigorate spleen to promote digestion		QXN041	W	0.009	0.63
Urticaceae	*Urtica fissa* E. Pritz	Whole plant	Herb	Field, Roadside, Behind the house	All year round	Using fresh herb, Soak in hot water	Eliminate wind and resolve dampness, moisten the lung and relieve cough		QXN092*	W	0.009	0.83
Verbenaceae	*Verbena officinalis* L	Whole plant	Herb	Roadside, Mountains and plains, Streamside	Summer	Decoction, Soak in hot water	Clear heat-toxin, soften hardness and dissipate masses		QXN006*	C/W	0.112	1.17
Violaceae	*Viola philippica* Cav	Whole plant	Herb	Field, Wilderness, Grasscluster, Brush	Spring, Summer	Sun drying, Decoction	Clear heat-toxin, soften hardness and dissipate masses		QXN048*	C	0.009	0.63
Vitaceae	*Causonis japonica* (Thunb.) Raf	Whole plant	Vine	Mountains and plains, Rinchu, Brush	Summer, Autumn	Sun drying, Decoction	Remove toxins, diuresis, soften hardness and dissipate masses		QXN060*	W	0.017	0.63
Zingiberaceae	*Zingiber officinale* Roscoe	Stem	Herb	Field	Autumn, Winter	Soak in hot water	Dissipate cold and eliminate dampness, moisten the lung and relieve cough		QXN067*	C	0.017	1.31

In the list, RFC relative frequency of citation; * = Chinese herbal medicine; “QXN+number” indicates specimens number of herbal tea plants; – indicates herbal tea plants with no specimens; C = Cultivation; W = Wild; C/W = Both cultivated and wild; (E) = Exotic plant

**Fig. 2:**
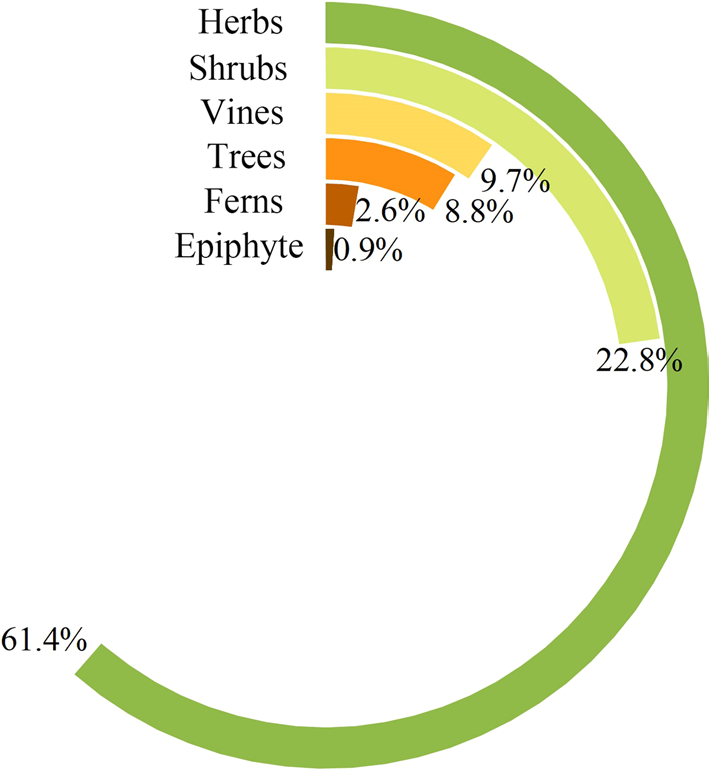
Life forms of herbal tea plants in Qianxinan Prefecture

The life forms were mainly herbs (accounting for 61.4%), followed by shrubs (22.8%), trees (8.8%) and vines (9.7%) (Fig. 2). Most of herbal tea plants are also found in Chinese Materia Medica, which proves that herbal tea plants have the same origin as medicine and food (Table 2). Local communities commonly adopted simple methods for herbal tea plant preparation, either by washing the plants for immediate use or by drying them for later utilization. These approaches require minimal investment, involve simple procedures and facilitate easy preservation, making herbal tea popular.

**Fig. 3:**
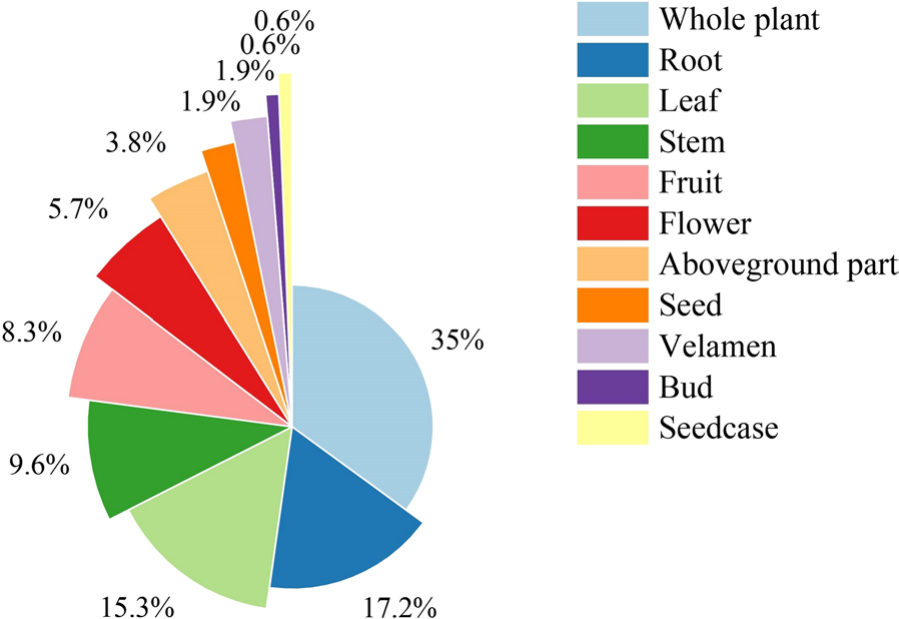
Use parts of herbal tea plants in Qianxinan Prefecture

Local people in Qianxinan utilized various parts of herbal tea plant resources, encompassing 11 different types: whole plants, roots, leaves, stems, fruits, flowers, aboveground parts, seeds, velamina, buds and seedcases. During the survey, a total of 157 occurrences of these parts were documented (Fig. 3). The whole plant and leaf parts of the plant are the preferred choices for daily herbal teas due to their accessibility and abundance. The whole plant was the most frequently mentioned (with 55 occurrences, 35%) which serves the dual purpose of reducing waste during the processing and increasing production of herbal tea plants. Roots, leaves, stems and fruits were also common utilization parts, with mentions ranging from 10 to 30 times. In contrast, flowers, aboveground parts, seeds, velamina, buds and seedcases were mentioned fewer than 10 times, indicating their relatively infrequent usage.

The collection of herbal tea plants can take place throughout the year, and different parts of the plant can be gathered for tea consumption at various times of the year (Table 2). Local inhabitants noted that different plant parts offer distinct flavors and nutritional benefits. For example, *Buddleja officinalis* was collected locally in February and March as special natural dyes and can be decocted for herbal tea. The tea prepared from flowers has a mellow and sweet taste. Fruits of *Pyracantha fortuneana* can serve as snacks, or be dried and soaked for a beverage with sweet and brisk taste. Different parts can be employed in diverse ways, either for medicinal purposes or for daily consumption, depending on their characteristics. The tea prepared from flowers often had a mellow and sweet taste, while the taste of tea made from leaves was relatively mellow and astringent.

The highest level of similarity in species-level herbal tea uses was observed between Zhenfeng and Ceheng, with a Jaccard index (JI) of 0.17. Conversely, the most significant differences were found between Zhenfeng and Wangmo, with a JI of 0.06. When examining the genus level, the utilization of herbal tea plant resources exhibited the highest similarity between Zhenfeng and Ceheng (JI = 0.18), while the similarity between Zhenfeng and Wangmo was lower (JI = 0.07).

### Health care effect of herbal tea plants

**Fig. 4:**
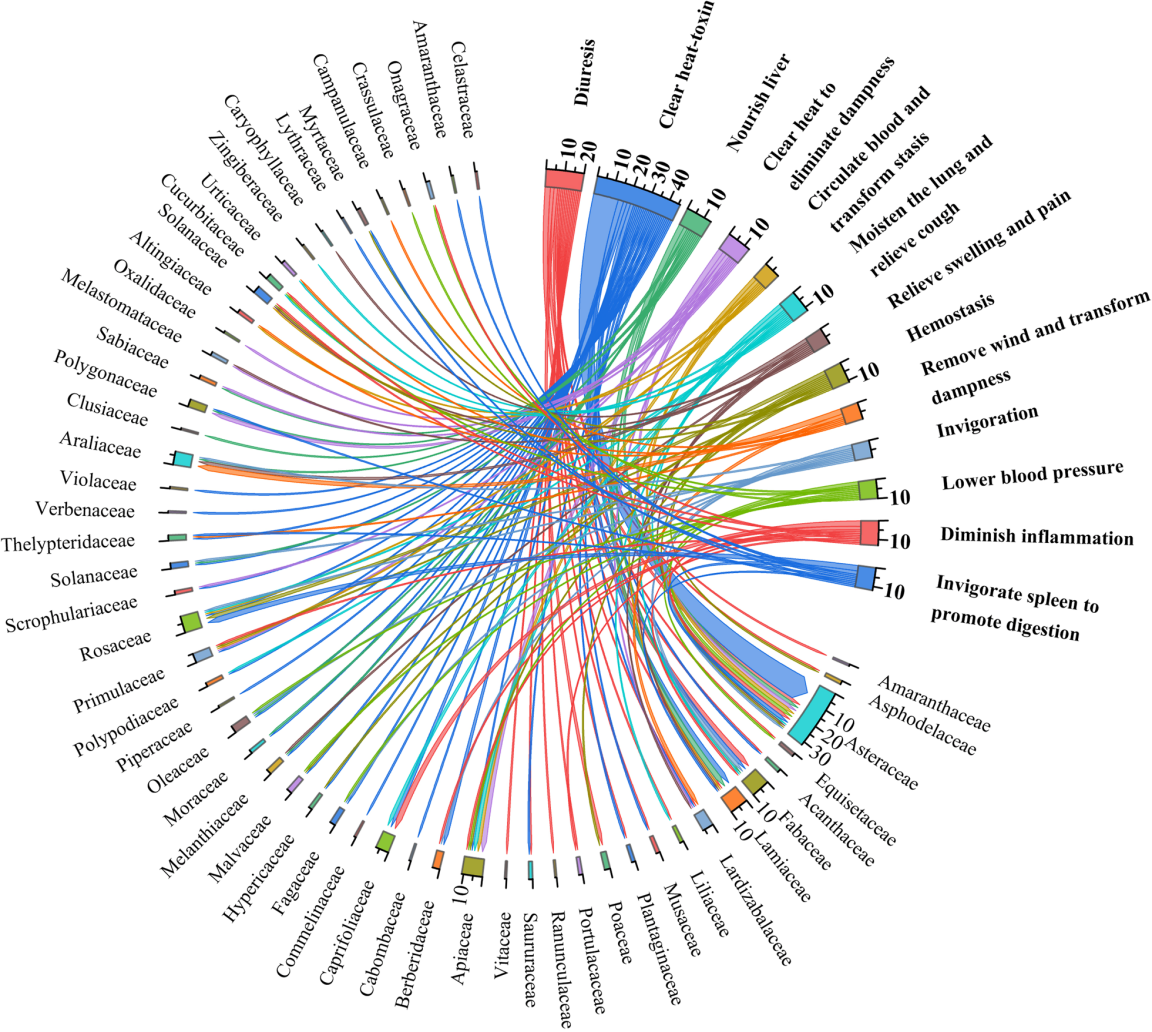
The efficacy of herbal tea and number of plants

The locals not only use herbal tea plants for making beverages but also incorporate them into their daily lives for various purposes such as seasoning, food coloring, and spices. The local population believes that herbal tea plants provide a wide range of health advantages effectively addressing a spectrum of 33 significant health disorders (Table 2). Clearing heat-toxin was the most commonly stated property, and there were 46 species that had this function and were mainly distributed in Asteraceae family (14 species, accounting for 30%). Diuresis and nourishing the liver were frequently cited by locals as the intended effects of consuming herbal tea, and these properties were associated with plants from the Fabaceae family (three species, accounting for 15%) and the Lamiaceae family (three species, accounting for 21%), respectively. Clearing heat and eliminating dampness (a total of 13 species, including three species from Apiaceae family), diminishing inflammation (13 species in total, three species of Caprifoliaceae), and invigorating the spleen to promote digestion (a total of 12 species, including three species from Rosaceae family) were also found to be prevalent within their respective plant families. Additionally, moistening the lung and relieve cough (a total of 12 species), hemostatis (a total of 11 species) and lowering blood pressure (a total of 10 species) were also frequently mentioned therapeutic benefits (Fig. 4).

### Resource availability hypothesis, versatility hypothesis and diversification hypothesis

**Fig. 5:**
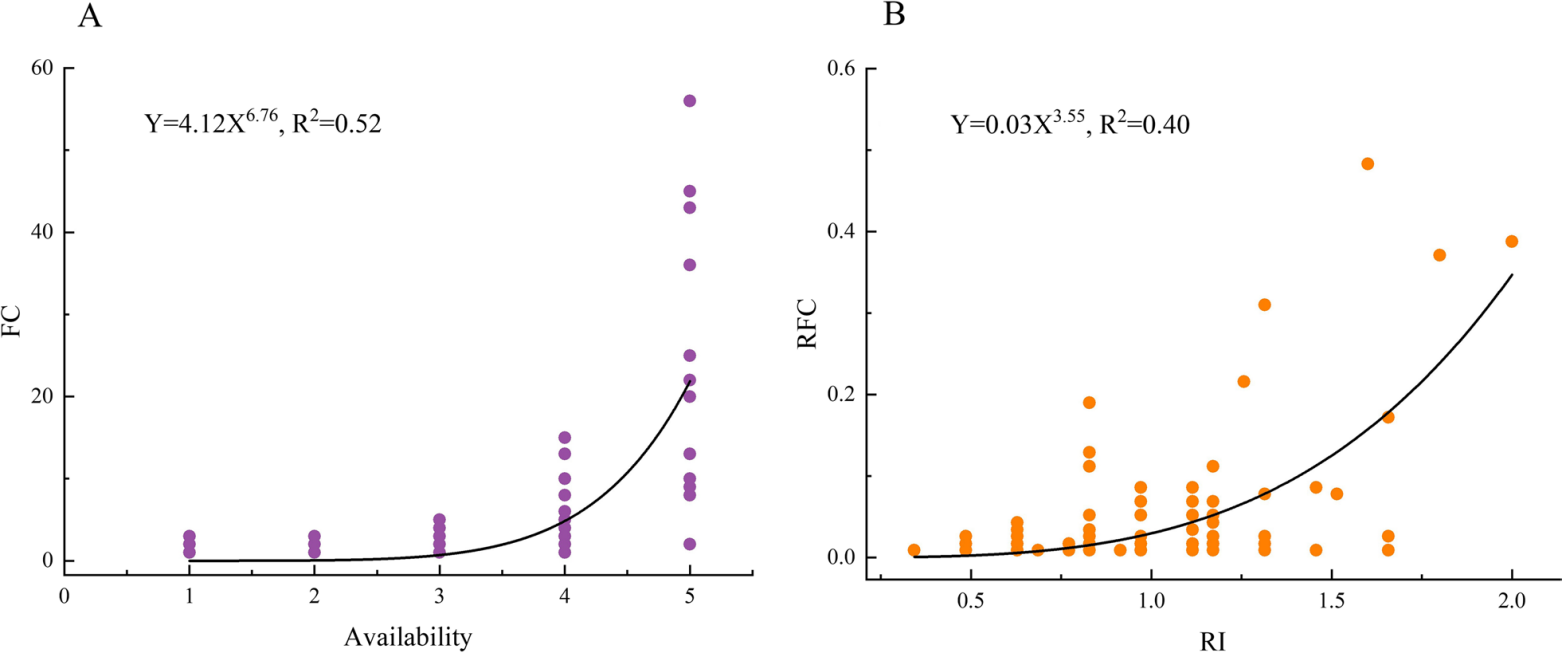
Correlation between herbal tea frequency of citation and plants availability and correlation between RFC and RI. **A** correlation between FC and Availability, **B** correlation between RI and RFC

According to the evaluation on the availability of recorded herbal tea plants, 14 species were most common plants (12%), 33 species were more common plants (29%), 30 species were common plants (26%), 22 species were less common plants (19%), and 15 species were uncommon plants (13%). The most common plants can be collected on the roadside, in front of and behind houses, where people live. The results showed that the frequency of citation of herbal tea plants in the study area was positively correlated with their availability (*r* = 0.72, *p* < 0.001) (Fig. 5A).

**Fig. 6:**
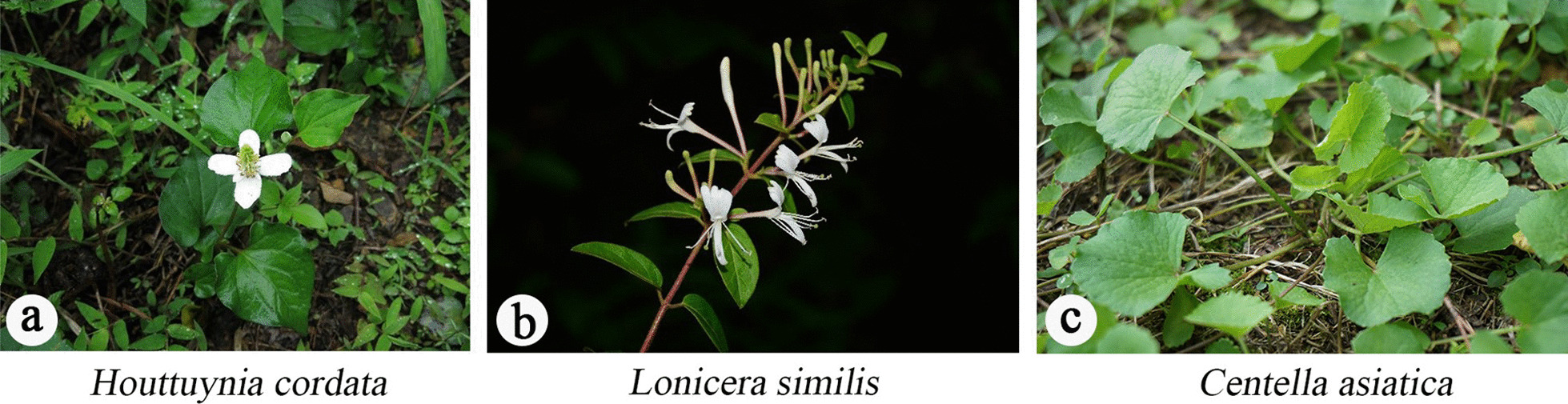
Plants cited frequently in Qianxinan. **a**: *Houttuynia cordata*, **b**: *Lonicera similis*, **c**: *Centella asiatica*

There were 55 herbal tea plants with relative importance higher than one, including *Centella asiatica*, *Lonicera similis*, *Taraxacum mongolicum*, *Artemisia argyi*, and *Houttuynia cordata*. General linear model showed a significant positive correlation between the relative importance of herbal tea plants and the frequency of mentions (*r* = 0.63, *p* < 0.001). *Houttuynia cordata* (RFC = 0.51, RI = 1.60), *Centella asiatica* (RFC = 0.38, RI = 2) and *Lonicera similis* (RFC = 0.37, RI = 1.8), were the most commonly used species for tea by locals (Fig. 6). Apparently, locals preferred to mention and use plants with higher versatility (Fig. 5B).

There were 11 exotic plants, accounting for 10.5% (Table 3), which were *Erigeron annuus*, *Bidens Pilosa*, *Crassocephalum crepidioides*, *Bidens bipinnata*, *Eclipta prostrata*, *Sonchus oleraceus*, *Oenothera rosea*, *Oenothera rosea*, *Amaranthus tricolor*, *Stellaria aquatica*, *Cyperus rotundus* and *Olanum pseudocapsicum*. Most of these plants were native to the Americas, followed by Asia, Africa and Europe. The RI values of 11 exotic plants were all above 0.6 (Table 3), and they were often used in the treatment of malaria. The number of native plants to treat gynecological diseases was similar to the number of exotic plants, but there were differences in the parts used. At present, half of the exotic herbal tea plants listed in the study area had been included in the books of Chinese Materia Medica (Table 2), and *Senna occidentalis* had been included “Processing standard of Chinese herbal slices in Guizhou Province (贵州省中药饮片炮制规范)”. In multipurpose herbal tea plants (RI > 1), 83% (46 species) were native plants. Although the RI value of exotic plants was relatively lower than native plants, they were rich in flavonoids, polyphenols, terpenoids and other compounds to treat certain diseases and were incorporated into the local medical system in the folk (Table 3).

**Table 3:** List of exotic herbal tea plants in Qianxinan

Plant name	RI	Place of origin	Peculiarity	References
*Amaranthus tricolor* L	1.0	India	Rich in protein, Zn, Ca and vitamins; in folk it has the advantages of relieving urine, supplementing calcium to coagulate blood and improving eyesight. The stems and leaves are eaten as vegetables; the leaves are mixed in various colors for viewing	[54]
*Bidens bipinnata* L	0.6	East Asia and North America	Rich in hyperoside, quercetin, rutin; it can be used as a folk medicine to treat sore throat, cold, malaria, abdominal pain and diarrhea	[55]
*Bidens pilosa* L	1.1	America	Rich in quercetin, hyperoside, taxfolin-7-o-rhamnoside; in folk the whole grass is decocted, to treat diabetes, and used externally to treat boils, snake bites, bruises and swelling pain	[56]
*Crassocephalum crepidioides* (Benth.) S. Moore	1.0	Tropical African	The stems are rich in dihydrocoumarin compounds, which have anti-dysentery effects; in folk it is often used to treat indigestion, and its young leaves are a delicious wild vegetable	[57]
*Cyperus rotundus* L	0.8	Asia	The main components are volatile oils, favonoids and sugars; it can be used as a medicine for gynecological diseases	[56]
*Eclipta prostrata* (L.) L	1.3	America	Rich in triterpenoid saponins, flavonoids; it has the effect of nourishing liver and kidney, cooling blood and stopping bleeding	[58]
*Erigeron annuus* (L.) Pers	1.0	Eastern America	Rich in vanillic acid, ferulic acid, 4-hydroxyacetophenone; in folk it is commonly used in the treatment of malaria, and it has the ability to clear heat-toxin,, and aid digestion	[56]
*Oenothera rosea* L'Her. ex Ait	1.0	Southern North America	Rich in kaempferol, ursolic acid, luteolin; in folk it is mainly used to decrease inflammation and pain caused by trauma	[56]
*Senna occidentalis* (Linnaeus) Link	1.5	Tropical America	Rich in β-sitosterol, daucosterol; in folk it is regarded as traditional medicine, and its efficacy is mainly evident in the treatment of malaria	[59]
*Solanum pseudocapsicum* L	1.1	Mexico, the Caribbean and South America	The whole plant is poisonous, and root has analgesic effect, and can be used to treat lumbar muscle strain	[60]
*Sonchus oleraceus* L	1.2	Europe and the Mediterranean	Rich in luteolin, apigenin and other glycosides; in folk it is commonly used to treat icteric hepatitis and is often eaten as a wild vegetable	[56]

### Preliminary exploration of *Centella asiatica* tea products

**Fig. 7:**
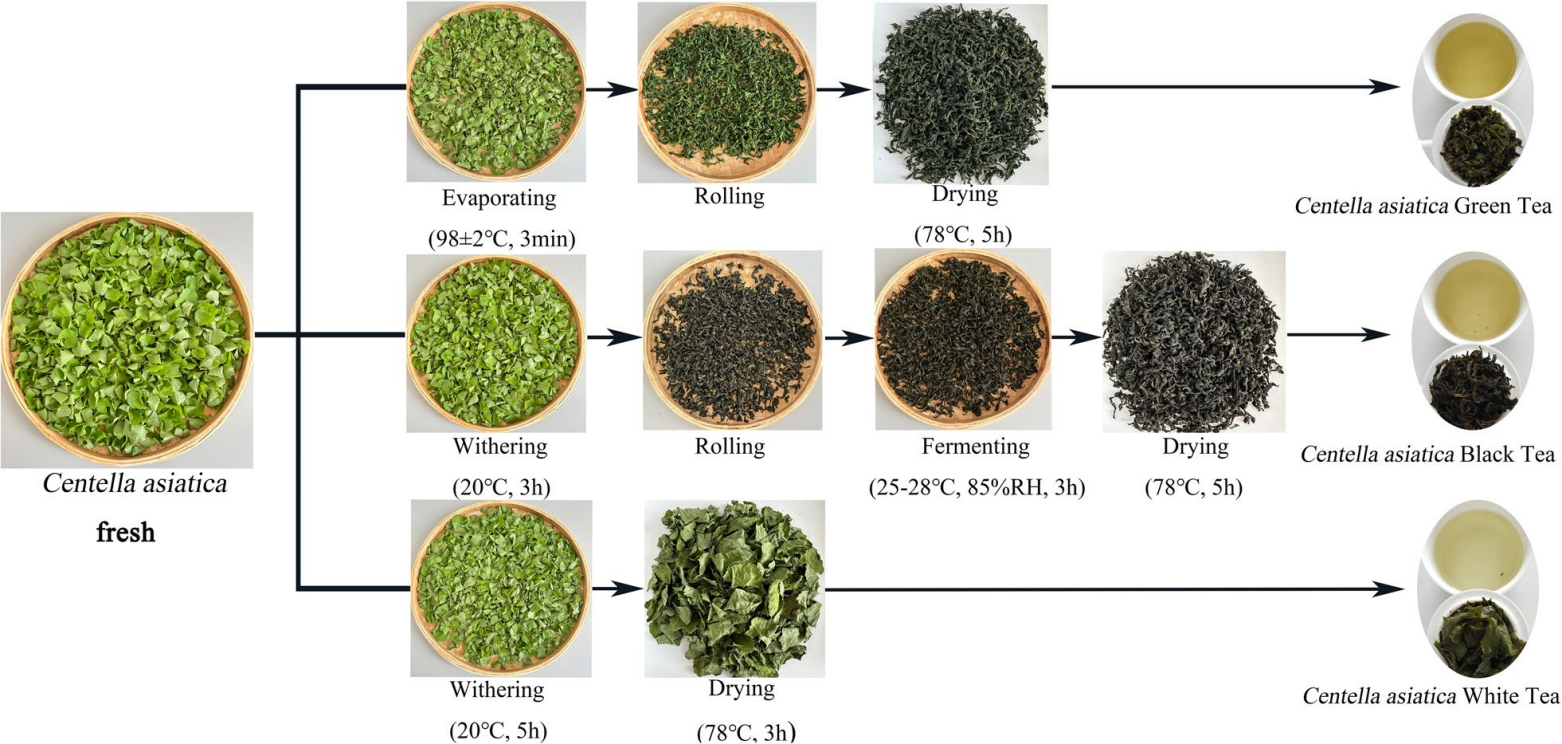
*Centella asiatica* tea production process and the soup

The RI value of *Centella asiatica* was one, and its types of uses covered food, medicine, and was usually used as tea drinks, with the effects of clearing heat-toxin and dampness,  eliminating swelling, promoting blood circulation and removing blood stasis. The tea was prepared by boiling it at a ratio of 1:50 tea for 5 mins, and the sensory quality of the tea was assessed based on attributes such as color, aroma, taste, leaf structure, and more. Regarding the appearance, the white *Centella asiatica* tea was distinctive in that it was not rolled, and the tea leaves remained intact and uniform. In contrast, the green and black tea processes required rolling, resulting in a more uniform appearance of tea leaves. In terms of the color of the tea, all three processes yielded a bright, yellow soup, with the white tea process producing a slightly lighter color. The taste of the tea varied across the different processes, with the green and black tea processes offering rich and diverse flavor profiles. Aroma differences were observed between the black tea process and the other two processes due to the fermentation (25-28 °C, 85% relative humidity-RH,  three hours) involved in the black tea process, which produced a stronger woody aroma. Regarding the leaf structure, the leaves were spread out uniformly under all three processing conditions. Notably, the leaves of the green tea were found to be softer compared to those of the other tea samples (Fig. 7, Table 4).

**Table 4:** Comparison of sensory quality of *Centella asiatica* tea

Tea sample	Appearance	Appearance of tea liquid	Taste	Aroma	Tea leaves
*Centella asiatica* Green Tea	Green and tight	Deep yellow	Strong and thick	Faint scent	Green and bright
*Centella asiatica* Black Tea	Brown and sturdy	Light yellow	Mellow and sweet	Woody	Bark and brown
*Centella asiatica* White Tea	Green and uniform	Apricot yellow	Sweet and smooth	Herb	Green and vivid

## Discussion

### Reasons for the use of herbal teas by local people

Our survey revealed that the local population has limited familiarity with the six major types of *Camellia* tea, which include green tea, black tea, dark tea, white tea, oolong tea and yellow tea. In contrast, there are a wide variety of herbal plants in Qianxinan, which are easily accessible and can be processed. The 114 species of herbal tea plants recorded in this survey are regarded as the traditional beverage plants of local people, of which 67 species are Chinese medicinal plants (58%). The medicinal functions of these plants are quite comprehensive, and their long-term use in the local area has proven effective in preventing, treating, and managing common illnesses. Asteraceae plants are known for their potent in clearing heat-toxin, as well as their ability to disperse blood stasis and alleviate pain. They also exhibit cooling effects on the blood and are frequently found in herbal form, making them convenient for collection. Whether ingested or applied topically, they can rapidly eliminate toxins from the body or the body's surface [61]. Rosaceae plants, on the other hand, are rich in saponins, which have a notable impact on blood vessels. They can reduce capillary permeability, minimize blood seepage, and serve as effective hemostatic agents [62]. The Apiaceae family is particularly noteworthy for its capacity to promote blood circulation, dispel blood stasis, alleviate itching and pain, and address issues related to dampness and cold. These plants excel in enhancing circulation and dispelling surface coldness [63]. *Argyreia pierreana* can be used both externally and internally, and it offers therapeutic benefits including clearing heat-toxin, treating traumatic injuries, as well as possessing anti-inflammatory and hemostatic properties [64]. *Perilla frutescens*, *Lophatherum gracile* and *Plantago asiatic*a have demonstrated diuretic effects [65–67]. *Mentha canadensis* is effective in treating gastrointestinal diseases and infectious diseases, with pharmacological activities that include hepatoprotective, anti-inflammatory and antioxidant properties [68].

Every ethnic group engages in subsistence activities to sustain its way of life, fulfill its most fundamental needs and support further development during their interactions with nature [69]. Local residents note that the harvesting time for herbal tea is spread throughout all seasons, and they collect the useful parts for consumption in accordance with the plant’s growth cycle. In spring and summer, when plants are in the vegetative growth stage, in addition to gathering parts for drinking, locals also harvest plant buds for use in dishes to meet their nutritional needs. During summer and autumn, when plants are in the reproductive growth stage, many are collected as whole plants, which can also be dried and used for medicinal purposes. In autumn and winter, when the aboveground parts of plants wither and the plants go dormant, the roots are collected and boiled since they contain higher levels of active ingredients. The consuming of herbal tea, using the traditional sun-drying method [70], and selling the excess in local markets to supplement their livelihoods are common practice among the Buyi people.

Frequently mentioned are plants that are readily available in proximity to people's living areas, making availability a significant factor influencing their preference for herbal tea. The resource availability hypothesis effectively elucidates the connection between the presence of herbal tea plants and their utilization by the local population across the study area.

### Processing of *Centella asiatica* tea product

Different processing methods yield three distinct types of *Centella asiatica* tea, each characterized by variations in color, aroma, taste and unique qualities. The color of tea soup is influenced by several factors during processing, with the quality characteristics of green tea primarily shaped by the fixation process. Fixation (enzyme inactivation), a critical step in green tea processing, serves to deactivate enzymatic activity within the leaves, thereby minimizing enzyme-driven oxidation and chlorophyll degradation. This preserves the distinctive characteristics of green tea leaves [71]. In our preliminary experiments, we observed that residual moisture on the surface of freshly cleaned *Centella asiatica* leaves causes them to stick together. Consequently, we employ an evaporating method without rapid leaf agitation during fixation. Evaporating durations within the range of 1–5 mins optimize the retention of DPPH (1,1-Diphenyl-2-picryl-hydrazyl) scavenging compounds in herbal tea [23, 26].

In the production of black tea, the key process is fermentation, which leads to moisture loss in the leaves during the initial withering phase. Following rolling, leaf cell disruption reaches 80%, initiating decomposition reactions primarily driven by macromolecules, as well as phenolic oxidation and chlorophyll degradation under enzymatic influence. These processes impart multi-layered taste characteristics to the fresh leaves [27, 72]. In our experiments, fermentation was conducted at 25–28 °C and 85% relative humidity for three hours, resulting in the transformation of leaf color from green to deep green. The *Centella asiatica* black tea produced exhibited a dark green appearance, with a light yellow infusion. Furthermore, polyphenols undergo gradual transformation during the extended withering process [25, 73], contributing to the development of a sweet and mellow taste profile.

Withering is a crucial stage in shaping the distinctive quality of white tea. The study revealed that the drying time required for the simple white tea processing is shorter in comparison to the processing times for green and black teas. This is primarily because the white tea process does not involve rolling the leaves; instead, the leaves are stretched, and there is a notable spacing between them. This allows the tea sample to dry faster and the processing time to be shortened. In sensory evaluation, the key parameters for assessing quality are appearance, aroma and taste, as these factors significantly influence consumer acceptance.

Overall*, Centella asiatica* white tea has few processing steps and basically imitated the traditional processing of the locals. The beneficial ingredients could be effectively preserved, while maintaining a sweet and smooth taste akin to traditional processing methods. The processing of *Centella asiatica* green tea and black tea may lead to a reduction in certain essential components of the leaves. However, it may also give rise to novel elements not present in traditional processing methods. Therefore, it is of significance to conduct in-depth investigations into the chemical composition of *Centella asiatica* tea under varying processing conditions. Such research holds great practical significance in optimizing the utilization of *Centella asiatica* resources.

The initial attempts have been made at *Centella asiatica* product processing also serve as a means to promote the diversification of beverages for urban residents and those residing in areas without *Centella asiatica* distribution.

### The use of herbal tea in modern society

Herbal teas are widely consumed in Yunnan, Guizhou, Guangxi, etc., offering both excellent taste and beneficial effects. With the increasing popularity of natural health teas, herbal teas have garnered the attention of many consumers. Examples include Ku Ding Tea (*Ilex kaushue*), honeysuckle tea (*Lonicera similis*), and Guangxi sweet tea (*Rubus chingii* var. *suavissimus*). The production of herbal teas has embraced innovation in traditional processing methods to cater to the diverse needs of the modern era. Leveraging the advantages of herbal tea plants, such as their pleasant taste, aroma and heat-clearing properties, and addressing the challenge of bitterness, well-prepared herbal teas can offer a delightful taste along with their therapeutic benefits [74]. In herbal tea processing, various techniques can be employed, including traditional drying and storage, as well as the creation of concentrated liquid or solid packaging formats to meet different requirements. It’s worth noting that the chemical composition content of the roots, stems, and leaves of herbal tea plants often differs. Therefore, making the best use of all parts of the plant can enhance the overall utilization rate of herbal tea plants. Currently, there is a need to reasonably, scientifically and comprehensively exploit the existing herbal tea plant resources and explore them from the perspective of plant biochemistry to develop new herbal tea plant products.

The processing and consumption of teas can also be adapted to the current fast-paced and convenient lifestyles of people by creating herbal tea beverages in teabag form or formulating them into liquid herbal tea beverages [75]. However, the proliferation of new herbal tea beverages like Wahaha and Nongfu Spring has led to the decline of traditional tea beverages. In an attempt to cater to the preferences of the majority of consumers, many manufacturers have started incorporating various food additives into herbal tea beverages, often overlooking the inherent nutritional value of the herbal tea plants themselves in pursuit of the commercial herbal tea beverage market [9]. The safety of consuming herbal tea beverages and the biological activity and toxicity of herbal tea plants are still subjects of ongoing research. The development of herbal tea beverages should be based on research into the pharmacological activities and constituents, ensuring their safety for consumption in both the pharmaceutical and food industries.

Furthermore, in alignment with the government’s initiative to revitalize rural areas, local governments should encourage agricultural cooperatives to collaborate with businesses and educational institutions for the cultivation of suitable herbal tea plant varieties in the region and establish cultivation parks. This not only fosters the growth of the local herbal tea industry but also advances the herbal tea sector as a whole, contributing to the livelihoods of local residents.

## Conclusions

As a commonplace daily beverage, herbal teas in Qianxinan Prefecture serve both medicinal and refreshing purposes for locals. The selected plants reflect accessibility, versatility and remedy augmentation needs as explained by established ethnobotanical hypotheses. Specifically, the resource availability hypothesis and versatility hypothesis provide insight into plant selection patterns, indicating that accessibility and versatility influence use. Additionally, exotic plants fill therapeutic gaps not addressed by native flora, aligning with the diversification hypothesis. This rich documented flora and associated indigenous knowledge is complemented by preliminary experimental efforts showcasing quality variances across processing methods for the widely used *Centella asiatica*. Green Tea, Black Tea, and White Tea products made from this species exhibit suitable sensorial attributes and utilization potential. Further research should build on these foundations to comprehensively investigate bioactivities, cultivation practices, innovative products and sustainable industry development. By elucidating plant utilization drivers, processing techniques and novel beverage prospects, this study makes valuable progress in understanding and promoting the under-researched domain of herbal teas and associated ethnomedicinal traditions in southwest China.

## Data Availability

All data generated or analyzed during this study are included in this published article.

## Abbreviations

RFC: Relative frequency of citation
RI: Relative importance
FC: Frequency of citation
NUC: Number of use categories
NT: Number of types
GB/T: Chinese national standards
T/CTSS: Association Standards by China Tea Science Society
RH: Relative humidity
DPPH: 1,1-Diphenyl-2-picryl-hydrazyl
QXN: Qianxinan
QXN: + number Specimens number of herbal tea plants
C: Cultivation
W: Wild
C/W: Both cultivated and wild
E: Exotic plant
